# Historical Perspective: Snail Control to Prevent Schistosomiasis

**DOI:** 10.1371/journal.pntd.0003657

**Published:** 2015-04-23

**Authors:** Charles H. King, David Bertsch

**Affiliations:** Center for Global Health and Diseases, Case Western Reserve University, Cleveland, Ohio, United States of America; George Washington University School of Medicine and Health Sciences, UNITED STATES

Effective interruption of the *Schistosoma* life cycle is essential to blocking the parasite’s transmission, and thus truly preventing human schistosomiasis over the long term. Our current mass treatment campaigns were expected to limit transmission by reducing environmental contamination with parasite eggs. However, the process of transmission has proven to be very focal and highly efficient, such that, in practice, mass drug treatment by itself has not been effective in lowering transmission in many affected areas. Alternative means for interruption of transmission are now being considered, including means to reduce or eliminate intermediate snail hosts from local habitats, and means to prevent water contamination through sanitation.

Before the introduction of safe oral drug therapy (e.g., praziquantel and oxamniquine), snail control for prevention of *Schistosoma* transmission was an important component of many regional schistosomiasis control programs (Fig 1). Now, following the 2012 London Declaration on Neglected Tropical Diseases and the recent recommendation by the World Health Assembly favoring local schistosomiasis elimination “where feasible” (WHA Resolution 65.21 [1]), interest has revived in supplementary non-drug means to achieve local elimination of *Schistosoma* transmission. This scoping historical review highlights some of the perceived strengths and weakness of transmission-interruption methods based on snail control, reexamining the results of programs of the 1950–1980s that aimed at control of *Bulinus* and *Biomphalaria* host snail species. In particular, we focus on the required inputs for effective control using the chemical molluscicide niclosamide, which is the 2-amino ethanol salt of 2', 5'-dichloro-4'-nitro salicylanilide, and which is sold as Bayluscide, Mollutox, and other names.

**Fig 1:**
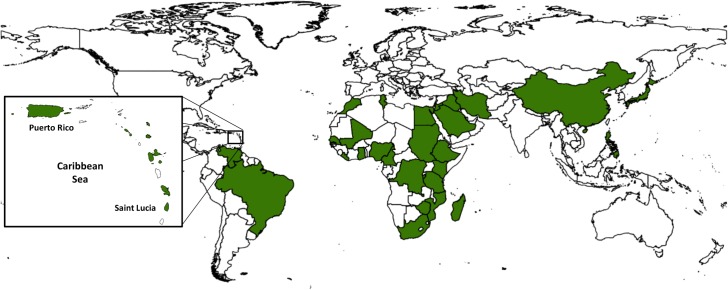
Countries reporting past use of molluscicide and/or snail habitat modification for control of *Schistosoma* transmission. Dark green shading indicates areas where long-term snail control interventions have been used as part of experimental or programmatic efforts to reduce or prevent *Schistosoma* transmission. The inset shows the island countries of the Caribbean basin where snail control has been used. See S1 Text for list of sources.

Application of chemical molluscicides, either by blanket or focal treatments, was the most common approach to snail control at *Schistosoma* transmission sites in Africa and the Americas during the 1950s–1970s. Although copper sulfate and sodium pentachlorophenate were in use in the 1950s, niclosamide became the compound most frequently studied in published reports after 1961 [2]. The perceived advantages of niclosamide, a newer compound, were its very low toxicity for humans and livestock and its ability to kill snails, their eggs, and cercariae at concentrations of <1 part per million (ppm, equivalent to 1 mg per liter concentration) [3]. Niclosamide was also relatively stable to ultraviolet radiation, with persistent lethal effects for up to 24 hours after application. Typical values for 90% snail mortality (LC_90_) are 0.25 ppm for *Biomphalaria pfeifferi*, 0.5 ppm for *Bulinus globosus*, and 0.25 ppm for *Bulinus tropicus*.

Perceived strengths and weaknesses of mollusciciding are listed in Tables 1 and 2, and a list of materials needed for a typical implementation of molluscicide application is provided in Table 3.

**Table 1:** Perceived advantages of using molluscicides.

1	Direct interruption of snail-to-human transmission
2	The desirable, but not essential, involvement of the community
3	A reasonable efficiency and cost of product
4	The simple equipment can also be used for the control of other vectors
5	While good supervision is essential, the methods of application are simple and do not require specialized operational schemes
6	Selection of foci for application can usually be based on the patterns of water use by the local population

Adapted from McCullough, et al. [4], and de Souza [5].

**Table 2:** Perceived disadvantages of using molluscicides.

1	Repeated reapplication is necessary, because snail eradication is often not possible.
2	Time demands of implementation and evaluation of control are greater than for MDA.
3	The impact on *Schistosoma* infection and morbidity is delayed relative to drug therapy.
4	Uniform dispersal and area coverage is difficult to achieve.
5	The cost of labor is foremost when doing repeated treatments.
6	Well-informed technical capacity is required to decide appropriate application.
7	Collateral molluscicide effects on amphibians and fish must be openly addressed and effectively minimized to meet public concerns about safety and environmental impact.

**Table 3:** Equipment typically needed for mollusciciding application.

**For flowing water sites:**
• Containers of 20, 60, or 120 liters fitted with a tap
• Spray pump or watering can
**For still water sites:**
• Spray pump or watering can
Evaluation is made 24 hr after application by direct observation of the snails in the breeding site.
**Other materials used in the field:**
Tape measure, string, long handled scoop, counter, paper, thermometer, stop watch (or watch), screen cages for placing sentinel snails, stakes, screen, molluscicides, polystyrene, equipment for application, container for dilution of the molluscicides, funnel, overalls, alcohol, gloves, mask
**Monitoring laboratory essentials:**
Gloves, alcohol, glass vials, trays, spring water, microscope, slides, forceps, transfer pipettes, biohazard containers

Adapted from de Souza [5].

For an effective control program, managers had to become familiar with the local snail genera, their preferred habitats, and the proper application of molluscicide to achieve its desired effect. As described by Palmer, et al. [6] in 1969,

“[T]he classic approach [was one] of centralized administration for control of vector-borne diseases, the field crews in [Puerto Rico] were directed by a project chief with his technical advisory group of biologists, sanitary engineers, and physicians. During the control phase of the project, a supervisor and five laborers with a vehicle worked full time…on the control of schistosomiasis.”

Operating procedures were developed to optimally target snails within their habitats at critical times during the year. Treatment frequency depended on whether the water habitat was static, slow- or fast-flowing, and/or seasonal. In 1961, Ayad [7] listed working priorities for mollusciciding as:

- making sure that the target snail is susceptible to achievable levels of molluscicide;
- surveying the area, dividing it into management units, counting resident snails, and keeping clear records of these findings;
- checking snail shedding rates for evidence of *Schistosoma* infection;
- studying the influence of agricultural practices and seasonal weather fluctuations on snail numbers;
- reviewing the available budget, personnel, logistics, and molluscicide;
- generating clear job descriptions, standard operating procedures [SOPs], and providing good supervision (both quality and quantity) by a separate audit team, with periodic reeducation of mollusciciding teams.

His teams in Egypt focused on individual transmission sites, areas of land reclamation, and those areas switching from seasonal to perennial irrigation. Priority was given to “radius control” within 500 m of human settlements. Snail numbers were monitored before and after treatments, then monthly after that, using the same personnel, with yearly assessments of snail numbers and human infection levels done at the same time of year. Slightly more than a decade later, Ayad concluded,

“The use of molluscicides still offers the greatest opportunity for the rapid control of schistosomiasis transmission. It also has the advantage of not requiring the active cooperation of the population as is the case in health education and mass chemotherapy. An additional advantage of considerable importance is that the use of molluscicides also controls the vectors of other trematode infections of domestic animals which are of the utmost economic importance, especially fascioliasis, thus contributing greatly to the protection of animal health. It should be stated, however, that because of the problem of reinfestation, the application of molluscicides, except in completely isolated places, has to be continually repeated and is, therefore, costly.” [8]

Ultimately, labor costs would prove to be the largest expense, creating a deterrent to the long-term continued use of mollusciciding. Where reported, labor costs accounted for 60% of the budget for local mollusciciding in Tanzania and in Southern Rhodesia (now Zimbabwe), 65% on St. Lucia [9], and > 60% in Puerto Rico and 36%–80% in Brazil [10].

The results of using mollusciciding alone for *Schistosoma* transmission control were mixed. In São Lourenço da Mata, Brazil, snail numbers fell to zero after three years but rebounded slightly in year six [11]. Infection in sentinel mice only fell to zero in year six, but an untreated comparison area also experienced a drop to zero the same year. A similar experience was reported in Puerto Rico [6] where, although the *Schistosoma* prevalence dropped more quickly and consistently in the island’s snail control areas, transmission was already decreasing island-wide. Human prevalence and intensity of *Schistosoma mansoni* dropped in both treated and untreated areas, suggesting that secular trends in other environmental factors were playing an influential role in the progressive elimination of *Schistosoma* transmission. A clear limitation of mollusciciding was the rapid re-emergence of snail numbers and of *Schistosoma* transmission within weeks to months after snail treatments. Pitchford [12], using test rodents, frequently found transmission at the end of each three month molluscicide treatment cycle. It became apparent that additional factors were responsible for the varying degrees of efficacy of niclosamide, including water flow rate, vegetation, temperature, water hardness/chemical makeup, and suspended sediment. Incomplete area coverage played a role in persistence and reintroduction of host snails.

Nevertheless, the effects of consistent multiyear niclosamide mollusciciding were clearly beneficial on St. Lucia [13], even where total elimination could not be achieved. In St. Lucia’s mollusciciding substudy region, prevalence dropped from 22% (in 1970) to 4.3% (1974–75) among children in high-risk villages, and from 4.4% to 2.2% in lower-risk villages following implementation of snail control alone. By contrast, in corresponding high- and low-risk untreated areas, prevalence remained high (22%–20%, and 8.5%–6.7%, respectively) during the same period. Other regions where mollusciciding and habitat modification were successful in reducing or eliminating *Schistosoma* infection included Iran, Tunisia, Morocco, St. Kitts, and the oases of Egypt (see S1 Text). Ultimately, like St. Lucia, many programs found that more frequent, highly focal applications could effectively reduce the number of transmitting (*Schistosoma-*infected) snails without having to completely suppress snail numbers. Informed timing of delivery, linked to peak transmission season and/or scheduled mass drug administration, has the potential to minimize mollusciciding and maximize its effects on transmission. However, proper implementation requires skilled supervision.

By the late 1970s and early 1980s, a wave of enthusiasm in policy circles about newly available oral treatment regimens for *Schistosoma* infections eclipsed the previous enthusiasm for snail control, in part because of the relatively slow impact of mollusciciding campaigns. From today’s perspective, given the published successes of snail control in a number of regions [8,14,15], this critique seems overly harsh. In a rebuttal to ASTMH President I. G. Kagan’s 1979 claim that that mollusciciding was “ineffective” for schistosomiasis control, J. D. Christie and colleagues [15] admitted that this might be true in a case “…where [molluscicides were] used in a haphazard manner, without appropriate precontrol studies.” They continued their defense of mollusciciding by adding,

"Obviously, in any situation in which pesticides are used without proper knowledge of the local epidemiology of any disease, then environmental impact on non-target species, lack of cost-effectiveness, development of target-species resistance, and failure to attain interruption of transmission may indeed occur. These are the real lessons to be learned from situations in which mollusciciding has been unsuccessful.”

Since that time, researchers have continued to experiment successfully with snail control by chemicals, predators, and by the plant-derived saponin molluscicide (Endod) as prevention of *Schistosoma* transmission. Endod has been specially promoted as a sustainable local product in East Africa [16]. Other research has demonstrated the feasibility and effectiveness of having local village health workers deliver mollusciciding, thus potentially reducing the need for extensive vertical infrastructure [17].

In retrospect, these historical data indicate that informed snail control can be an effective means for reducing local *Schistosoma* transmission. When properly implemented, snail control’s effects are likely to favorably complement those of modern-day mass drug delivery programs, resulting in much improved prevention of *Schistosoma* infection and reinfection. For the next phase of schistosomiasis control, formal trials of such integrated intervention will be welcome.

## Supporting Information

S1 Text Selected bibliography on schistosomiasis, molluscicides, and snail control. (DOCX)
