# Supplementary material for: Historical Perspective: Snail Control to Prevent Schistosomiasis
Source: PLoS Negl Trop Dis. 2015 Apr 23;9(4):e0003657. doi: 10.1371/journal.pntd.0003657 (PMC4408102; doi:10.1371/journal.pntd.0003657)
Supplement: S1 Text — (DOCX) [file pntd.0003657.s001.docx]

**Supporting Information- Document S1**

**Selected Bibliography on Snail Hosts and Molluscicidal Agents for Control of Schistosomiasis Mansoni and Schistosomiasis Haematobia—in chronological order**

1. Mozley A (1952) Molluscicides. London: H.K. Lewis & Co.

2. Mozley A (1955) Sites of Infection: Unstable Areas as Sources of Parasitic Diseases: Schistosomiasis and Fascioliasis. London: H.K. Lewis & Co. 86 p.

3. Chernin E, Michelson EH, Augustine DL (1956) Studies on the biological control of schistosome-bearing snails. I. The control of *Australorbis glabratus* populations by the snail, *Marisa cornuarietis*, under laboratory conditions. Am J Trop Med Hyg 5: 297-307.

4. McCullough FS (1957) The seasonal density of populations of *Bulinus (Physopsis) globosus* and *B. forskalii* in natural habitats in Ghana. Ann Trop Med Parasitol 51: 235-248.

5. McCullough FS (1957) The distribution of human schistosomiasis and the potential snail hosts in Ghana. West Afr Med J 6: 87-97.

6. McCullough FS (1957) A preliminary note on the degree of compatibility between *Schistosoma haematobium* and its bulinid vectors. West Afr Med J 6: 98-100.

7. Webbe G, Msangi AS (1958) Observations on three species of *Bulinus* on the east coast of Africa. Ann Trop Med Parasitol 52: 302-314.

8. Wright WH, Dobrovolny CG, Berry EG (1958) Field trials of various molluscicides (chiefly sodium pentachlorophenate) for the control of aquatic intermediate hosts of human bilharziasis. Bull World Health Organ 18: 963-974.

9. McCullough FS (1959) The susceptibility and resistance of *Bulinus (Physopsis) globosus* and *Bulinus (Bulinus) truncatus* *rohlfsi* to two strains of *Schistosoma haematobium* in Ghana. Bull World Health Organ 20: 75-85.

10. Foster R, Teesdale C, Poulton GF (1960) Trials with a new molluscicide. Bull World Health Organ 22: 543-548.

11. Clarke VD, Shiff CJ, Blair DM (1961) The control of snail hosts of bilharziasis and fascioliasis in Southern Rhodesia. Bull World Health Organ 25: 549-558.

12. Gillet J, Bruaux P (1961) [Laboratory trials of the new molluscicides Bayer 73 and ICI 24223]. Bull World Health Organ 25: 509-517.

13. Gönnert R (1961) Results of laboratory and field trials with the molluscicide Bayer 73. Bull World Health Organ 25: 483-501.

14. Paulini E, Chaia G, de FJ (1961) Trials with the molluscicides Rhodiacid and Bayer 73. Bull World Health Organ 25: 706-709.

15. Pesigan TP, Hairston NG (1961) The effect of snail control on the prevalence of *Schistosoma japonicum* infection in the Philippines. Bull World Health Organ 25: 479-482.

16. Shiff CJ (1961) Trials with a new molluscicide, Bayer 73, in Southern Rhodesia. Bull World Health Organ 25: 533-542.

17. Shiff CJ, Garnett B (1961) The short-term effects of three molluscicides on the microflora and microfauna of small, biologically stable ponds in Southern Rhodesia. Bull World Health Organ 25: 543-547.

18. Webbe G (1961) Laboratory and field trials of a new molluscicide, Bayer 73, in Tanganyika. Bull World Health Organ 25: 525-531.

19. Crossland NO (1962) A mud-sampling technique for the study of the ecology of aquatic snails, and its use in the evaluation of the efficacy of molluscicides in field trials. Bull World Health Organ 27: 125-133.

20. Gillet J, Bruaux P (1962) Laboratory and field testing of Bayluscide (Bayer 73). Pflanzen-schutz-Nachrichten Bayer 15: 70-74.

21. Mandahl-Barth G (1962) Key to the identification of east and central African freshwater snails of medical and veterinary importance. Bull World Health Organ 27: 135-150.

22. McMullen DB, Buzo ZJ, Rainey MB, Francotte J (1962) Bilharziasis control in relation to water resources development in Africa and the Middle East. Bull World Health Organ 27: 25-40.

23. Meyling AH, Schutte CH, Pitchford RJ (1962) Some laboratory investigations on Bayer 73 and ICI 24223 as molluscicides. Bull World Health Organ 27: 95-98.

24. Webbe G (1962) The transmission of *Schistosoma haematobium* in an area of Lake Province, Tanganyika. Bull World Health Organ 27: 59-85.

25. Crossland NO (1963) A large-scale experiment in the control of aquatic snails by the use of molluscicides on a sugar estate in the northern region of Tanganyika. Bull World Health Organ 29: 515-524.

26. Ritchie LS, Berrios-Duran LA, Frick LP, Fox I (1963) Molluscicidal qualities of Bayluscide (Bayer 73) revealed by 6-hour and 24-hour exposures against representative stages and sizes of *Australorbis glabratus*. Bull World Health Organ 29: 281-286.

27. Crossland NO (1964) The pest status and control of the tadpole shrimp, *Triops granarius,* and of the snail, *Lanistes ovum,* in Swaziland rice fields. Journal of Applied Ecology 2: 115-120.

28. Gamet A, Brottes H, Mvogo L (1964) [Preliminary experiments in the control of bilharziasis vectors in the ponds of pisciculture station in the Cameroons]. Bull Soc Pathol Exot Filiales 57: 118-124.

29. Webbe G (1964) Control of transmission of *Schistosoma mansoni* (Sambon) in the Mirongo River. East Afr Med J 41: 508-520.

30. Webbe G, Sturrock RF (1964) Laboratory tests of some new molluscicides in Tanganyika. Ann Trop Med Parasitol 58: 234-239.

31. Hairston NG (1965) An analysis of age-prevalence data by catalytic models. A contribution to the study of bilharziasis. Bull World Health Organ 33: 163-175.

32. Webbe G (1965) Natural trends in snail populations in relation to control of bilharziasis in East Africa. East Afr Med J 42: 605-613.

33. WHO (1965) Molluscicide screening and evaluation. Bull World Health Organ 33: 567-581.

34. Dawood IK, Dazo BC, Farooq M (1966) Large-scale application of Bayluscide and sodium pentachlorophenate in the Egypt-49 project area. Evaluation of relative efficacy and comparative costs. Bull World Health Organ 35: 357-367.

35. Farooq M, Hairston NG, Samaan SA (1966) The effect of area-wide snail control on the endemicity of bilharziasis in Egypt. Bull World Health Organ 35: 369-375.

36. Harrison AD (1966) The effects of Bayluscid on gastropod snails and other aquatic fauna in Rhodesia. Hydrobiologia 28: 371-384.

37. Pitchford RJ (1966) Findings in relation to schistosome transmission in the field following the introduction of various control measures. S Afr Med J 40 Suppl: 3-16.

38. Arfaa F, Bijan H, Farahmandian I (1967) Present status of urinary bilharziasis in Iran. Trans R Soc Trop Med Hyg 61: 358-367.

39. Boyce CB, Tieze-Dagevos JW, Larman VN (1967) The susceptibility of *Biomphalaria glabrata* throughout its life-history to N-tritylmorpholine. Bull World Health Organ 37: 13-21.

40. Crossland NO (1967) Field trials to evaluate the effectiveness of the molluscicide N-tritylmorpholine in irrigation systems. Bull World Health Organ 37: 23-42.

41. Foster R (1967) Schistosomiasis on an irrigated estate in East Africa. 2. Epidemiology. J Trop Med Hyg 70: 159-168.

42. Gönnert R (1967) Experiences in the control of bilharziasis with molluscicides. Ann Soc Belges Med Trop Parasitol Mycol 47: 195-203.

43. Harrison AD, Mason MH (1967) The effects on the fauna of natural waters of surveillance treatment with Bayluscide in Rhodesia. Hydrobiologia 29: 149-155.

44. Jobin WR, Unrau GO (1967) Chemical control of *Australorbis glabratus*. Public Health Rep 82: 63-71.

45. Marking LL, Hogan JW (1967) Toxicity of Bayer 73 to fish. In: Service FaW, editor. Washington: US Department of the Interior.

46. Berrios-Duran LA, Ritchie LS, Wessel HB (1968) Field screening tests on molluscicides against *Biomphalaria glabrata* in flowing water. Bull World Health Organ 39: 316-320.

47. Ferguson FF, Palmer JR, Jobin WR (1968) Control of schistosomiasis on Vieques Island, Puerto Rico. Am J Trop Med Hyg 17: 858-863.

48. Jobin WR (1968) Rationale for selecting molluscicides for bilharzia control programs. Public Health Rep 83: 594-596.

49. Jobin WR (1968) Economics of the application of molluscicides to flowing water. Bull World Health Organ 38: 322-323.

50. Webbe G (1968) Quantitative studies of intermediate host populations in the transmission of schistosomes. Proc R Soc Med 61: 455.

51. Farooq M (1969) Pre-control investigations in bilharziasis. J Trop Med Hyg 72: 14-18.

52. Massoud J, Chu KY, Arfaa F (1969) Field trials of Bayluscide, sodium pentachlorophenate and copper sulphate in standing waters in Iran. Ann Trop Med Parasitol 63: 189-194.

53. Palmer JR, Colon AZ, Ferguson FF, Jobin WR (1969) The control of schistosomiasis in Patillas, Puerto Rico. Public Health Rep 84: 1003-1007.

54. Arfaa F, Farahmandian I, Sahba GH, Bijan H (1970) Progress towards the control of bilharziasis in Iran. Trans R Soc Trop Med Hyg 64: 912-917.

55. Arfaa F, Farahmandian I, Soleimani M (1970) Evaluation of the effect of mass chemotherapy with niridazole as a method of bilharziasis control in Iran. Trans R Soc Trop Med Hyg 64: 130-133.

56. Barnish G (1970) The control of bilharziasis by the use of molluscicides. Cent Afr J Med: Suppl:22-27.

57. Fenwick A (1970) The development of snail control methods on an irrigated sugar-cane estate in northern Tanzania. Bull World Health Organ 42: 589-596.

58. Fenwick A, Lidgate HJ (1970) Attempts to eradicate snails from impounded water by the use of N-tritylmorpholine. Bull World Health Organ 42: 581-588.

59. Hira PR (1970) Aspects of the spread and control of schistosomiasis in Ibadan, Nigeria. West Afr Med J Niger Pract 19: 180-183.

60. Jobin WR (1970) Population dynamics of aquatic snails in three farm ponds of Puerto Rico. Am J Trop Med Hyg 19: 1038-1048.

61. Jobin WR (1970) Control of *Biomphalaria glabrata* in a small reservoir by fluctuation of the water level. Am J Trop Med Hyg 19: 1049-1054.

62. Jobin WR, Ferguson FF, Palmer JR (1970) Control of schistosomiasis in Guayama and Arroyo, Puerto Rico. Bull World Health Organ 42: 151-156.

63. Levine ND (1970) Integrated control of snails. Am Zool 10: 579-582.

64. Shiff CJ (1970) The role of molluscicides in bilharzia control. S Afr Med J 44: 167-168.

65. Barbosa FS, Pinto R, Souza OA (1971) Control of schistosomiasis mansoni in a small north east Brazilian community. Trans R Soc Trop Med Hyg 65: 206-213.

66. Duke BO, Moore PJ (1971) The control of *Schistosoma haematobium* in West Cameroon. Trans R Soc Trop Med Hyg 65: 841-843.

67. Zaki AA (1971) The effect of systematic application of Bayluscide on controlling bilharziasis. East Afr Med J 48: 218-227.

68. Farringer JE (1972) The determination of the acute toxicity of Rotenone and Bayer 73 to selected aquatic organisms. La Crosse, Wisconsin: University of Wisconsin. 32 p.

69. Fenwick A (1972) Effect of a control programme on transmission of *Schistosoma mansoni* on an irrigated estate in Tanzania. Bull World Health Organ 47: 325-330.

70. Fenwick A, Jorgensen TA (1972) The effect of a control programme against *Schistosoma mansoni* on the prevalence and intensity of infection on an irrigated sugar estate in northern Tanzania. Bull World Health Organ 47: 579-586.

71. Barnish G, Sturrock RF (1973) Letter: Aerial application of a molluscicide to a marsh. Trans R Soc Trop Med Hyg 67: 610-611.

72. Clarke VV, Blair DM, Weber MC (1973) The Mayfield experiment: an attempt to eliminate bilharziasis from a small community by repeated treatment of the infected people combined with intensive snail control. Cent Afr J Med: 14-22.

73. Gilles HM, Abdel-Aziz Zaki A, Soussa MH, Samaan SA, Soliman Soliman S, et al. (1973) Results of a seven year snail control project on the endemicity of *Schistosoma haematobium* infection in Egypt. Ann Trop Med Parasitol 67: 45-65.

74. Macdonald F, Clarke Vde V, Gaddie P, Atkinson G (1973) Report on a large-scale attempt at control of bilharziasis by combined mass treatment and intensive snail control. Cent Afr J Med 19: 22-32.

75. Shiff CJ (1973) The value of incidence for the assessment of schistosomiasis control; a study in Southern Rhodesia. Bull World Health Organ 48: 409-414.

76. Shiff CJ, Clarke Vde V, Evans AC, Barnish G (1973) Molluscicide for the control of schistosomiasis in irrigation schemes: a study in Southern Rhodesia. Bull World Health Organ 48: 299-307.

77. Sturrock RF (1973) Field studies on the transmission of *Schistosoma mansoni* and on the bionomics of its intermediate host, *Biomphalaria glabrata*, on St. Lucia, West Indies. Int J Parasitol 3: 175-194.

78. Sturrock RF (1973) Control of *Schistosoma mansoni* transmission: strategy for using molluscicides on St. Lucia. Int J Parasitol 3: 795-801.

79. Sturrock RF, Barnish G (1973) The aerial application of molluscicides with special reference to schistosomiasis control. Bull World Health Organ 49: 283-285.

80. Thomas JD (1973) Schistosomiasis and control of molluscan hosts of human schistosomes with particular reference to possible self-regulatory mechanisms. Adv Parasitol 11: 307-394.

81. Choudhry AW (1974) Seven years of snail control at MWEA Irrigation Settlement, Kenya: results and costs. East Afr Med J 51: 600-609.

82. Highton RB, Choudhry AW (1974) The cost evaluation of mollusciciding operations on five irrigation schemes in Kenya. East Afr Med J 51: 180-193.

83. Lyons GR (1974) Schistosomiasis in north-western Ghana. Bull World Health Organ 51: 621-632.

84. Sturrock RF, Barnish G, Upatham ES (1974) Snail findings from an experimental mollusciciding programme to control *Schistosoma mansoni* transmission in St. Lucia. International Journal of Parasitology 4: 231-240.

85. Ayad N (1976) Snail control and some significant control projects, review. Egypt J Bilharz 3: 129-155.

86. Duke BO, Moore PJ (1976) The use of a molluscicide in conjunction with chemotherapy to control *Schistosoma haematobium* at the Barombi Lake foci in Cameroon. III. Conclusions and costs. Tropenmed Parasitol 27: 505-508.

87. Duke BO, Moore PJ (1976) The use of a molluscicide in conjunction with chemotherapy to control *Schistosoma haematobium* at the Barombi Lake foci in Cameroon. II. Urinary examination methods, the use of niridazole to attack the parasite in man, and the effect on transmission from man to snail. Tropenmed Parasitol 27: 489-504.

88. Duke BO, Moore PJ (1976) The use of a molluscicide, in conjunction with chemotherapy, to control *Schistosoma haematobium* at the Barombi Lake foci in Cameroon. I. The attack on the snail hosts, using N-tritylmorpholine, and the effect on transmission from snail to man. Tropenmed Parasitol 27: 297-313.

89. Amin MA, Fenwick A (1977) The development of an annual regimen for blanket snail control on the Gezira Irrigated Area of the Sudan. Ann Trop Med Parasitol 71: 205-212.

90. Klumpp RK, Chu KY (1977) Ecological studies of *Bulinus rohlfsi*, the intermediate host of *Schistosoma haematobium* in the Volta Lake. Bull World Health Organ 55: 715-730.

91. Rosenfield PL, Smith RA, Wolman MG (1977) Development and verification of a schistosomiasis transmission model. Am J Trop Med Hyg 26: 505-516.

92. Amin AH, Fenwick A (1978) The control of snails on a small scale. Trop Doct 8: 8-12.

93. Chu KY (1978) Trials of ecological and chemical measures for the control of *Schistosoma haematobium* transmission in a Volta Lake village. Bull World Health Organ 56: 313-322.

94. Jordan P, Barnish G, Bartholomew RK, Grist E, Christie JD (1978) Evaluation of an experimental mollusciciding programme to control *Schistosoma mansoni* transmission in St Lucia. Bull World Health Organ 56: 139-146.

95. Jobin WR (1979) Cost of snail control. Am J Trop Med Hyg 28: 142-154.

96. Shiff CJ, Coutts WC, Yiannakis C, Holmes RW (1979) Seasonal patterns in the transmission of *Schistosoma haematobium* in Rhodesia, and its control by winter application of molluscicide. Trans R Soc Trop Med Hyg 73: 375-380.

97. Barnish G, Christie JD, Prentice MA (1980) *Schistosoma mansoni* control in Cul de Sac Valley, Saint Lucia. I. A two-year focal surveillance-mollusciciding programme for the control of *Biomphalaria glabrata*. Trans R Soc Trop Med Hyg 74: 488-492.

98. Christie JD, Prentice MA, Barnish G (1980) Control of schistosomiasis by mollusciciding. Am J Trop Med Hyg 29: 323-324.

99. Jordan P, Christie JD, Unrau GO (1980) Schistosomiasis transmission with particular reference to possible ecological and biological methods of control. A review. Acta Trop 37: 95-135.

100. Jordan P, Cook JA, Bartholomew RK, Grist E, Auguste E (1980) *Schistosoma mansoni* control in Cul de Sac Valley, Saint Lucia. II. Chemotherapy as a supplement to a focal mollusciciding programme. Trans R Soc Trop Med Hyg 74: 493-500.

101. McCullough FS, Gayral P, Duncan J, Christie JD (1980) Molluscicides in schistosomiasis control. Bull World Health Organ 58: 681-689.

102. Prentice MA, Barnish G (1980) Granule formulations of molluscicides for use in developing countries. Ann Trop Med Parasitol 74: 45-51.

103. Barbosa FS, Costa DP (1981) A long-term schistosomiasis control project with molluscicide in a rural area of Brazil. Ann Trop Med Parasitol 75: 41-52.

104. Barnish G, Prentice MA (1981) Lack of resistance of the snail *Biomphalaria glabrata* after nine years of exposure to Bayluscide. Trans R Soc Trop Med Hyg 75: 106-107.

105. Chu KY, Klumpp RK, Kofi DY (1981) Results of three years of cercarial transmission control in the Volta Lake. Bull World Health Organ 59: 549-554.

106. Chu KY, Vanderburg JA, Klumpp RK (1981) Transmission dynamics of miracidia of *Schistosoma haematobium* in the Volta Lake. Bull World Health Organ 59: 555-560.

107. Fenwick A, Cheesmond AK, Amin MA (1981) The role of field irrigation canals in the transmission of *Schistosoma mansoni* in the Gezira Scheme, Sudan. Bull World Health Organ 59: 777-786.

108. McCullough FS (1981) Biological control of the snail intermediate hosts of human *Schistosoma* spp.: a review of its present status and future prospects. Acta Trop 38: 5-13.

109. Prentice MA, Jordan P, Bartholomew RK, Grist E (1981) Reduction in transmission of *Schistosoma mansoni* by a four-year focal mollusciciding programme against *Biomphalaria glabrata* in Saint Lucia. Trans R Soc Trop Med Hyg 75: 789-798.

110. Barnish G (1982) Evaluation of chemotherapy in the control of *Schistosoma mansoni* in Marquis Valley, Saint Lucia. II. Biological results. Am J Trop Med Hyg 31: 111-115.

111. Barnish G, Jordan P, Bartholomew RK, Grist E (1982) Routine focal mollusciciding after chemotherapy to control *Schistosoma mansoni* in Cul de Sac valley, Saint Lucia. Trans R Soc Trop Med Hyg 76: 602-609.

112. Barnish G, Prentice MA (1982) Predation of the snail *Biomphalaria glabrata* by freshwater shrimps in St. Lucia, West Indies. Ann Trop Med Parasitol 76: 117-120.

113. Machado PA (1982) The Brazilian program for schistosomiasis control, 1975-1979. Am J Trop Med Hyg 31: 76-86.

114. Andrews P, Thyssen J, Lorke D (1983) The biology and toxicology of molluscicides, Bayluscide. Pharmacol Ther 19: 245-295.

115. Evans AC (1983) Control of schistosomiasis in large irrigation schemes by use of niclosamide. A ten-year study in Zimbabwe. Am J Trop Med Hyg 32: 1029-1039.

116. Goll PH, Lemma A, Duncan J, Mazengia B (1983) Control of schistosomiasis in Adwa, Ethiopia, using the plant molluscicide endod (Phytolacca dodecandra). Tropenmed Parasitol 34: 177-183.

117. Saladin B, Saladin K, Holzer B, Dennis E, Hanson A, et al. (1983) A pilot control trial of schistosomiasis in central Liberia by mass chemotherapy of target populations, combined with focal application of molluscicide. Acta Trop 40: 271-295.

118. Goll PH, Wilkins HA, Marshall TF (1984) Dynamics of *Schistosoma haematobium* infection in a Gambian community. II. The effect on transmission of the control of *Bulinus senegalensis* by the use of niclosamide. Trans R Soc Trop Med Hyg 78: 222-226.

119. Polderman AM (1984) Cost-effectiveness of different ways of controlling intestinal schistosomiasis: a case study. Soc Sci Med 19: 1073-1080.

120. Thomas JD, Tait AI (1984) Control of the snail hosts of schistosomiasis by environmental manipulation: a field and laboratory appraisal in the Ibadan area, Nigeria. Philos Trans R Soc Lond B Biol Sci 305: 201-253.

121. el Gaddal AA (1985) The Blue Nile Health Project: a comprehensive approach to the prevention and control of water-associated diseases in irrigated schemes of the Sudan. J Trop Med Hyg 88: 47-56.

122. Sleigh AC, Mott KE, Hoff R, Barreto ML, Mota EA, et al. (1985) Three-year prospective study of the evolution of Manson's schistosomiasis in north-east Brazil. Lancet 2: 63-66.

123. Tameim O, Abdu KM, el Gaddal AA, Jobin WR (1985) Protection of Sudanese irrigation workers from schistosome infections by a shift to earlier working hours. J Trop Med Hyg 88: 125-130.

124. Tameim O, Zakaria ZB, Hussein H, el Gaddal AA, Jobin WR (1985) Control of schistosomiasis in the new Rahad Irrigation Scheme of Central Sudan. J Trop Med Hyg 88: 115-124.

125. Korte R, Schmidt-Ehry B, Kielmann AA, Brinkmann UK (1986) Cost and effectiveness of different approaches to schistosomiasis control in Africa. Trop Med Parasitol 37: 149-152.

126. Madsen H, Rohde R, Maiga AS (1986) Trials on focal molluscicide application in larger irrigation canals and lakes in Mali. Trop Med Parasitol 37: 22-24.

127. McCullough F (1986) Snail control in relation to a strategy for reduction of morbidity due to schistosomiasis. Trop Med Parasitol 37: 181-184.

128. Tanaka H, Yasuraoka K, McCullough FS (1986) Parameters used for assessment of molluscicides. Jpn J Exp Med 56: 189-194.

129. Anonymous (1987) Report of an independent evaluation mission on the national control programme in Egypt-1985. Trans R Soc Trop Med Hyg 81: 1-57.

130. Chandiwana SK (1987) Community water-contact patterns and the transmission of *Schistosoma haematobium* in the highveld region of Zimbabwe. Soc Sci Med 25: 495-505.

131. Jurberg P (1987) Why it is difficult to control *Biomphalaria glabrata,* the vector snail of schistosomiasis. Mem Inst Oswaldo Cruz 82 Suppl 4: 203-207.

132. Bolton P (1988) Schistosomiasis control in irrigation schemes in Zimbabwe. J Trop Med Hyg 91: 107-114.

133. Brinkmann UK, Werler C, Traore M, Korte R (1988) The costs of schistosomiasis control in a Sahelian country. Trop Med Parasitol 39: 175-181.

134. Chandiwana SK, Taylor P, Chimbari M, Ndhlovu P, Makura O, et al. (1988) Control of schistosomiasis transmission in newly established smallholder irrigation schemes. Trans R Soc Trop Med Hyg 82: 874-880.

135. Werler C (1989) Efficiency of focal molluscicide treatment against schistosomiasis reinfection in an irrigation scheme and in a small dams area in Mali. Preliminary communication. Trop Med Parasitol 40: 234-236.

136. Woolhouse ME, Chandiwana SK (1989) Spatial and temporal heterogeneity in the population dynamics of *Bulinus globosus* and *Biomphalaria pfeifferi* and in the epidemiology of their infection with schistosomes. Parasitology 98: 21-34.

137. Greer GJ, Mimpfoundi R, Malek EA, Joky A, Ngonseu E, et al. (1990) Human schistosomiasis in Cameroon. II. Distribution of the snail hosts. Am J Trop Med Hyg 42: 573-580.

138. Gundersen SG, Birrie H, Torvik HP, Scherbaum H (1990) Control of *Schistosoma mansoni* in the Blue Nile Valley of western Ethiopia by mass chemotherapy and focal snail control: a primary health care experience. Trans R Soc Trop Med Hyg 84: 819-825.

139. Webbe G, el Hak S (1990) Progress in the control of schistosomiasis in Egypt 1985-1988. Trans R Soc Trop Med Hyg 84: 394-400.

140. Woolhouse ME, Chandiwana SK (1990) The epidemiology of schistosome infections of snails: taking the theory into the field. Parasitol Today 6: 65-70.

141. Woolhouse ME, Chandiwana SK (1990) Population dynamics model for *Bulinus globosus*, intermediate host for *Schistosoma haematobium*, in river habitats. Acta Trop 47: 151-160.

142. al-Madani AA (1991) Problems in the control of schistosomiasis in Asir Province, Saudi Arabia. J Community Health 16: 143-149.

143. de Souza CP, Mendes NM (1991) [Repopulation of breeding habitats of *Biomphalaria glabrata* after treatment with niclosamide]. Rev Inst Med Trop Sao Paulo 33: 297-302.

144. Hofkin BV, Stryker GA, Koech DK, Loker ES (1991) Consumption of *Biomphalaria glabrata* egg masses and juveniles by the ampullariid snails *Pila ovata*, *Lanistes carinatus* and *Marisa cornuarietis*. Acta Trop 49: 37-44.

145. Coura Filho P, Rocha RS, de Lima ECMF, Katz N (1992) A municipal level approach to the management of schistosomiasis control in Peri-Peri, MG, Brazil. Rev Inst Med Trop Sao Paulo 34: 543-548.

146. Coura-Filho P, Mendes NM, de Souza CP, Pereira JP (1992) The prolonged use of niclosamide as a molluscicide for the control of *Schistosoma mansoni*. Rev Inst Med Trop Sao Paulo 34: 427-431.

147. de Lima e Costa MF, Rocha RS, Coura Filho P, Katz N (1993) A 13-year follow-up of treatment and snail control in an area endemic for *Schistosoma mansoni* in Brazil: incidence of infection and reinfection. Bull World Health Organ 71: 197-205.

148. Engels D, Ndoricimpa J, Gryseels B (1993) Schistosomiasis mansoni in Burundi: progress in its control since 1985. Bull World Health Organ 71: 207-214.

149. Meyer-Lassen J, Daffalla AA, Madsen H (1994) Evaluation of focal mollusciciding in the Rahad Irrigation Scheme, Sudan. Acta Trop 58: 229-241.

150. Pieri OS, Goncalves JF, Sarquis O (1995) Repeated focal mollusciciding for snail control in a sugar-cane area of northeast Brazil. Mem Inst Oswaldo Cruz 90: 535-536.

151. Souza CP (1995) Molluscicide control of snail vectors of schistosomiasis. Mem Inst Oswaldo Cruz 90: 165-168.

152. Greer GJ, Tchounwou PB, Takougang I, Monkiedje A (1996) Field tests of a village-based mollusciciding programme for the control of snail hosts of human schistosomes in Cameroon. Trop Med Int Health 1: 320-327.

153. Lima e Costa MF, Guerra HL, Pimenta FG, Jr., Firmo JO, Uchoa E (1996) [Evaluation of a program for the control of schistosomiasis in municipalities located on the basin of the Sao Francisco River, Minas Gerais, Brazil]. Rev Soc Bras Med Trop 29: 117-126.

154. Amazigo UO, Anago-Amanze CI, Okeibunor JC (1997) Urinary schistosomiasis among school children in Nigeria: consequences of indigenous beliefs and water contact activities. J Biosoc Sci 29: 9-18.

155. Sarquis O, Pieri OS, dos Santos JA (1997) Effects of Bayluscide WP 70 on the survival and water-leaving behaviour of Biomphalaria straminea, snail host of schistosomiasis in northeast Brazil. Mem Inst Oswaldo Cruz 92: 619-623.

156. Bagalwa M, Baluku B (1998) [Monthly variations in the level of infestation and the potential for transmission of *Biomphalaria pfeifferi* in 2 aquatic systems in Lwiro, Democratic Republic of Congo]. Med Trop (Mars) 58: 372-374.

157. Khallaayoune K, Madsen H, Laamrani H (1998) Evaluation of three methods to control *Bulinus truncatus*, the intermediate host of *Schistosoma haematobium* in an irrigation scheme, Tessaout-Amont, Morocco. Acta Trop 69: 51-63.

158. Pieri OS, Barbosa CS, Moza PG (1998) Schistosomiasis control based on repeated chemotherapy in a rural village of the sugar-cane zone in northeast Brazil. Mem Inst Oswaldo Cruz 93 Suppl 1: 259-264.

159. Pointier JP, Giboda M (1999) The case for biological control of snail intermediate hosts of *Schistosoma mansoni*. Parasitol Today 15: 395-397.

160. Laamrani H, Khallaayoune K, Boelee E, Laghroubi MM, Madsen H, et al. (2000) Evaluation of environmental methods to control snails in an irrigation system in Central Morocco. Trop Med Int Health 5: 545-552.

161. Douglas PT (2001) The control of *Schistosoma haematobium* in Kenya using molluscicide [MSc thesis]. London UK: The London School of Hygiene and Tropical Medicine. 49 p.

162. Sturrock RF, Diaw OT, Talla I, Niang M, Piau JP, et al. (2001) Seasonality in the transmission of schistosomiasis and in populations of its snail intermediate hosts in and around a sugar irrigation scheme at Richard Toll, Senegal. Parasitology 123 Suppl: S77-89.

163. Giovanelli A, Silva CL, Medeiros L, Vasconcellos MC (2002) The molluscicidal activity of niclosamide (Bayluscide WP70(R)) on *Melanoides tuberculata* (Thiaridae), a snail associated with habitats of *Biomphalaria glabrata* (Planorbidae). Mem Inst Oswaldo Cruz 97: 743-745.

164. Dawson VK (2003) Environmental fate and effects of the lampricide Bayluscide: a review. J Great Lakes Res 29: 475-492.

165. Morley NJ, Irwin SW, Lewis JW (2003) Pollution toxicity to the transmission of larval digeneans through their molluscan hosts. Parasitology 126 Suppl: S5-26.

166. Scholefield RJ, Bergstedt RA, Bills TD (2003) Relation of concentration and exposure time to the efficay of niclosamide against larval sea lampreys (*Petromyzon marinus*). J Great Lakes Res 29: 493-499.

167. Takougang I, Meli J, Angwafo F, 3rd (2006) Field trials of low dose Bayluscide on snail hosts of schistosome and selected non-target organisms in sahelian Cameroon. Mem Inst Oswaldo Cruz 101: 355-358.

168. Arijo AG, Qureshi TA, Pathan ZA (2007) A studies on chemical control of *S. mansoni* intermediate host. Pak J Biol Sci 10: 2606-2608.

169. Takougang I, Meli J, Wabo Pone J, Angwafo F, 3rd (2007) Community acceptability of the use of low-dose niclosamide (Bayluscide), as a molluscicide in the control of human schistosomiasis in Sahelian Cameroon. Ann Trop Med Parasitol 101: 479-486.

170. Yang GJ, Li W, Sun LP, Wu F, Yang K, et al. (2010) Molluscicidal efficacies of different formulations of niclosamide: result of meta-analysis of Chinese literature. Parasit Vectors 3: 84.

171. Yang GJ, Sun LP, Hong QB, Zhu HR, Yang K, et al. (2012) Optimizing molluscicide treatment strategies in different control stages of schistosomiasis in the People's Republic of China. Parasit Vectors 5: 260.

172. Kariuki HC, Madsen H, Ouma JH, Butterworth AE, Dunne DW, et al. (2013) Long term study on the effect of mollusciciding with niclosamide in stream habitats on the transmission of schistosomiasis mansoni after community-based chemotherapy in Makueni District, Kenya. Parasit Vectors 6: 107.

173. Barakat RMR (2013) Epidemiology of schistosomiasis in Egypt: Travel through time: Review. Journal of Advanced Research 4: 425-432.
